# Analysis of Metabolites in White Flowers of *Magnolia Denudata* Desr. and Violet Flowers of *Magnolia Liliiflora* Desr.

**DOI:** 10.3390/molecules23071558

**Published:** 2018-06-27

**Authors:** Chang Ha Park, Soo-Yun Park, Sook Young Lee, Jae Kwang Kim, Sang Un Park

**Affiliations:** 1 Department of Crop Science, Chungnam National University, 99 Daehak-Ro, Yuseong-gu, Daejeon 34134, Korea; parkch804@gmail.com; 2 National Institute of Agricultural Sciences, Rural Development Administration, Wanju-gun, Jeonbuk 54875, Korea; psy22@korea.kr; 3 Regional Innovation Center for Dental Science & Engineering, Chosun University, 309 Pilmun-daero, Dong-gu, Gwangju 501-759, Korea; koreanseedbank@gmail.com; 4 Division of Life Sciences and Bio-Resource and Environmental Center, Incheon National University, Incheon 406-772, Korea

**Keywords:** *Magnolia denudata* Desr., *Magnolia liliiflora* Desr., flowers, metabolic analysis

## Abstract

A total of seven phenolics and 44 metabolites was profiled in white flowers of *Magnolia denudata* and violet flowers of *Magnolia liliiflora* using high-performance liquid chromatography (HPLC), electrospray ionization-mass spectrometry (ESI-MS), and gas chromatography time-of-flight mass spectrometry (GC-TOFMS). Seven phenylpropanoid compounds were identified in white flowers by liquid chromatography mass spectrometry (LC-MS). An HPLC analysis showed that phenylpropanoid accumulation in violet flowers was 1.48 times higher than that in white flowers. Furthermore, superoxide dismutase (SOD)-like activity and 1,1-diphenyl-2-picrylhydrazyl (DPPH) radical scavenging activity were determined to investigate the antioxidant properties of secondary metabolites in different flowers. Violet flowers showed higher SOD-like and DPPH activity than white flowers. In addition, anti-inflammatory activity measured using a nitric oxide assay was higher in violet flowers than in white flowers. Our results provide valuable information on the relationship between primary and secondary metabolites, and synergistic antioxidant and anti-inflammatory properties derived from phenolic compounds in different colored flowers.

## 1. Introduction

**Figure 1:**
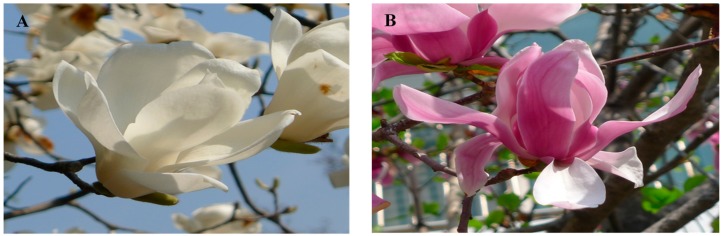
Two different colored flowers. (**A**) White flowers of *M. denudata*; (**B**) Violet flowers of *M. liliiflora*.

*Magnolia*, the largest genus in the *Magnoliaceae* family with more than 128 species, is widely distributed in East and Southeast Asia. These plants typically produce a large number of highly fragrant, cup shaped, and conspicuous flowers [1,2]. In Asian herbal medicine, *Magnolia* flowers and bark have been used to treat symptoms such as headache, thrombotic stroke, gastrointestinal disorders, anxiety, and allergies [3,4]. The use as a medicinal product is based on a substantial number of different phytochemicals with anxiolytic [4], antiplatelet [5], antipyretic [6], anti-inflammatory [6], antidiarrheal [7], antioxidant [7], anti-stomach ulcer [8], and anti-allergic properties [9]. *Magnolia denudata* Desr. and *Magnolia liliiflora* Desr. are commonly used as ornamental plants due to their attractive flowers; however, they are also utilized in traditional medicine (Figure 1). Traditionally, *Magnolia denudata* Desr. has been used as analgesic agent treating headache caused by sinusitis, nasal congestion, and allergic rhinitis [10,11]. Similarly, *Magnolia liliiflora* Desr has been used for treatment of headache, lumbago, and rhinitis [12]. 

Flavonoids are phenolic compounds commonly found in plants, and can be categorized in six classes: anthocyanins, isoflavones, flavones, flavanones, flavanols, and flavanonols [13]. The flavonoids play a crucial role for the development, growth, and pigment production of plants [14,15]. Moreover, these compounds are important for the protection of plants against environmental biological and physical stressors, including pathogen assault, insect herbivory, wounding, ultraviolet (UV) radiation, excessive light, and water deficiency [14,15,16]. Anthocyanins, belonging to flavonoids, are plant pigments exhibiting blue, pink, orange, violet, or red color in plant organs. Although a vast number of anthocyanins occurs naturally, six particular anthocyanidins (including petunidin, malvidin, peonidin, pelargonidin, cyanidin, and delphinidin) are the most common pigment compounds in vascular plants [17,18,19]. Studies on the biological activities of flavonoids showed a diversity of biological characteristics, such as antimicrobial, anti-inflammatory, anticarcinogenic, and antiviral, and antioxidant activities [20,21].

Metabolomic profiling has provided vital information for systematic botany by qualitative and quantitative measurement of various cellular metabolites [22,23,24]. Such measurements have been performed successfully using chromatography-mass spectrometry systems, which have facilitated the detection of numerous metabolites and their full or partial isolation [25]. Among these systems, liquid chromatography mass spectrometry (LC-MS) has been employed most frequently to detect metabolites of relatively high-molecular-weight, which cannot be analyzed using gas chromatography (GC) [26]. The method of GC time-of-flight mass spectrometry (GC-TOFMS) operates under fast scan time, and produces high mass accuracy and resolution for the detection of metabolites of relatively low molecular weight (<1000 Da^1^) [27,28].

RAW 264.7 is the murine macrophage cell line used for screening natural products for biological activity and for predicting their potential effect on primary cells or in vivo. The RAW 264.7 cell line response is used to determine the effective biological activity and regarded as a reflection of the potential human *de novo* response [29].

To our knowledge, to date, no study has investigated the relationship between primary and secondary metabolites in white flowers of *M. denudata* and violet flowers of *M. liliiflora*. The aim of this study was to elucidate the relationship between large numbers of primary and secondary metabolites in white and violet flowers using HPLC, LC-MS, and GC-TOFMS in combination with chemometrics. Furthermore, we investigate the relationship between these metabolites and antioxidant activity.

## 2. Results and Discussion

### 2.1. LC-MS and HPLC Analysis

A total of seven phenolic compounds, including three flavonoids and four phenolic acids, was identified in the flowers of *M. denudata*. by LC-MS analysis (Table 1). Three flavonoids (rutin, quercetin, and kaempferol) were identified as negatively monocharged molecular ions, and four phenolic acids (4-hydroxybenzoic acid, chlorogenic acid, caffeic acid, and *p*-coumaric acid) were identified as negatively monocharged molecular ions (Figure S1 and Table 1).

Seven of the phenolic compounds were identified and quantitated in the different colored Magnolia flowers by comparison of retention time, spike tests, and external standard calibration curves using HPLC (Table 1). Violet flowers contained higher concentrations of total flavonoids (17.31 ± 2.39 mg/g dry weight (wt.)) which is 1.48 times the concentration found in white flowers (11.71 ± 0.46 mg/g (wt.)). Furthermore, a comparison of individual phenolic compounds revealed that violet flowers contained higher amounts of 4-hydroxybenzoic acid, chlorogenic acid, caffeic acid, *p*-coumaric acid, and rutin than those of white flowers, respectively. On the other hand, the unknown compound, showed the largest peak area, was detected (Figure S1). 

### 2.2. Total Phenolic and Anthocyanin Contents

Total phenolic and anthocyanin contents of white and violet flowers, respectively, were measured using the Folin-Denis assay and total anthocyanin content assay. Violet flowers contained 1.20 times higher amount of total phenolics than that in white flowers. Furthermore, anthocyanin concentrations were 3.71 times higher in violet flowers than in white flowers (Table 2).

### 2.3. GC-TOFMS Analysis

A total of 44 hydrophilic metabolites identified in white flowers of *M. denudata* and violet flowers of *M. liliiflora* was quantitated using selected ions. After normalization based on signal intensity of an internal standard, a principal component analysis (PCA) was performed to investigate on differences in quantitative metabolite profiles between the two flowers. A component score plot of the PCA results shows an overview of the differences between the two different colored flowers and a component pattern plot was constructed to examine the correlations between the 44 metabolites (Figure S2). The samples were differentiated by the two highest ranked components, with the first component explaining 70.69% of the total variance, thereby separating the metabolite profiles of violet and white flowers. This effect is predominantly due to amino acids and sugars, as the respective loading was negative for most carbohydrates, except for trehalose, raffinose, and mannitol, and positive for most amino acids, except for 4-aminobutyric acid and glutamic acid. The results of PCA showed higher carbohydrate concentrations in violet flowers than in white flowers, and higher amino acid concentrations in white flowers than in violet flowers.

**Figure 2:**
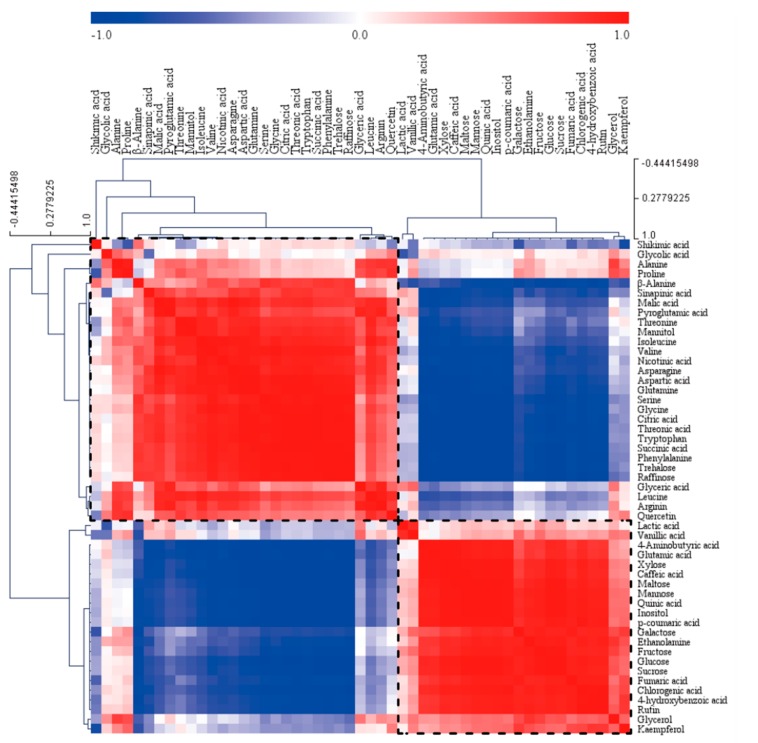
Correlation matrix of 51 metabolites from white flowers of *M. denudata* and violet flowers of *M. liliiflora*. Each square indicates the Pearson’s correlation coefficient of a pair of compounds, with the correlation coefficient indicated by the intensity of the red and blue colors. Dashed-line boxes indicate the 51 metabolites were clustered by an HCA using the Pearson’s correlation and average linkage clustering.

Furthermore, hierarchical cluster analysis (HCA) was performed using Pearson’s correlation results in order to examine the relationships between metabolites of white flowers of *M. denudata* and violet flowers of *M. liliiflora*. The results of HCA revealed the degree of correlation among 51 metabolites, which were identified and quantitated by GC-TOFMS and HPLC. A total of 51 metabolites was clustered together by the HCA using the Pearson’s correlation and average linkage clustering method, and marked by dashed-line boxes (Figure 2). One group contained the highest amount of amino acids (except 4-aminobutyric acid and glutamic acid), whereas the other group contained all sugars (except mannitol, trehalose, and raffinose), and phenolic compounds (except sinapic acid and quercetin). These results confirm the loadings found in the PCA. Most Pearson’s correlation coefficients between sugars and phenolic compounds exceeded 0.8. A significantly positive relationship was observed between sucrose, which is involved in phenylpropanoid biosynthesis, and phenolics, including 4-hydroxybenzoic acid (*r* = 0.90532, *p* = 0.013), chlorogenic acid (*r* = 0.9397, *p* = 0.0053), caffeic acid (*r* = 0.94274, *p* = 0.0048), *p*-coumaric acid (*r* = 0.95664, *p* = 0.0028), rutin (*r* = 0.91394, *p* = 0.0108), and quinic acid (*r* = 0.98037, *p* = 0.0006). Among phenolics, vanillic acid was positively correlated with caffeic acid, quinic acid, *p*-coumaric acid, chlorogenic acid, 4-hydroxybenzoic acid, rutin, and kaempferol, and sinapinic acid was positively correlated with quercetin and vanillic acid. Furthermore, quercetin was positively correlated with kaempferol (Figure S3).

**Figure 3:**
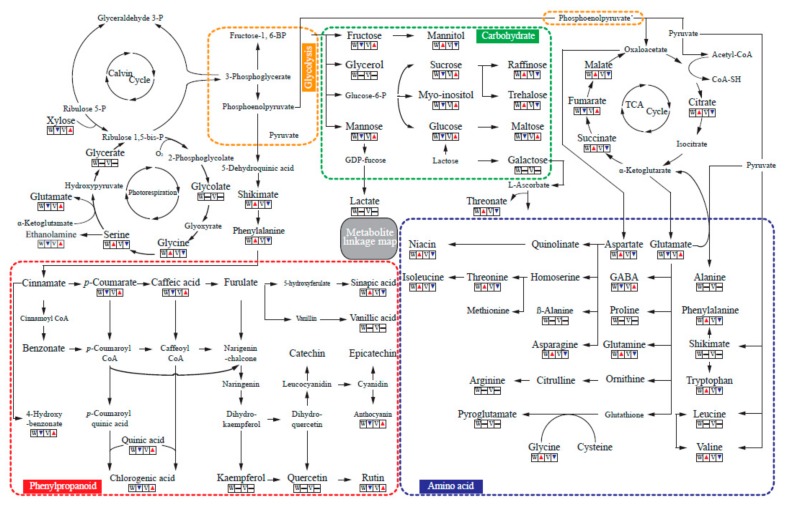
A metabolic linkage map comparing primary and secondary metabolites of white flowers of *M. denudata* and violet flowers of *M. liliiflora*. The up arrow (▲) indicates a significantly higher mean value of the metabolite (at *p* < 0.05). The down arrow (▼) indicates a significantly lower mean value of the metabolite (at *p* < 0.05). The horizontal bar (–) indicates that no significant difference was found (at *p* < 0.05).

A metabolite linkage map was produced based on a total of 44 metabolites identified by GC-TOFMS, and 7 phenolics quantified by HPLC, to investigate metabolic similarities between white and violet flowers of *M. denudata* (Figure 3 and Figure S4). Carbohydrates are molecular compounds consisting of oxygen (O), hydrogen (H), and carbon (C), predominantly used as energy and carbon sources, signaling molecules, osmotic agents, and protectants against certain stressor in plants [30]. In both flowers carbohydrates, including fructose, xylose, glucose, galactose, mannose, sucrose, maltose, trehalose, mannitol, glycerol, and raffinose, were the most abundant metabolites. The total amount of carbohydrates in violet flowers was 1.74-fold higher than that in white flowers, indicating higher energy and carbon demands for the production of phenolic compounds. Specifically, the concentrations of fructose, glucose, mannose, sucrose, maltose, and xylose, were higher in violet flowers. In contrast, the levels of mannitol, raffinose, and trehalose were higher in white flowers (Figure S4). Amino acids are organic nitrogen compounds formed by the assimilation of inorganic nitrogen and binding to a carbon skeleton to produce amino acids such as asparagine, aspartate, glutamine, and glutamate [31]. In the present study, a total of 20 different amino acids was identified in both flowers. The total amino acid concentration in white flowers was, on average, 1.30 times that of violet flowers. Specifically, the individual levels of the amino acids valine, serine, isoleucine, glycine, threonine, phenylalanine, asparagine, glutamine, tryptophan, and aspartate were higher in the white flowers. In contrast, two of the amino acids, 4-Aminobutyric acid, and glutamate, were found in higher concentrations in violet flowers (1.42-fold, and 1.42-fold, respectively). The greater abundance of asparagine (1.27-fold), aspartate (1.60-fold), and glutamine (1.24-fold), which is involved in nitrogen metabolism, in the white flowers reflected the higher levels of other amino acids. Furthermore, a total of four photorespiratory intermediates was identified in both flowers. White flowers, however, contained larger amounts of glycine (1.74-fold) and serine (1.40-fold). White flowers also contained larger amounts of most TCA cycle intermediates, including citrate (1.33-fold), succinate (2.69-fold), and malate (1.40-fold), which were associated with the higher concentrations of higher amino acids, including alanine, GABA, aspartate, tyrosine, and asparagine, compared to violet flowers. However, the amount of fumarate was 1.20-fold higher in violet than in white flowers. Regarding phenolic compounds, higher concentrations of phenolics such as chlrogenic acid, caffeic acid, 4-hydroxybenzoic acid, *p*-coumaric acid, rutin, and anthocyanins were found in violet flowers. In contrast, white flowers contained the higher concentration of sinapic acid. Anthocyanin, which is present in higher concentrations in violet flowers, presumably necessitates higher amounts of precursors or intermediates, including *p*-coumaric acid and caffeic acid. 

### 2.4. In-Vitro Antioxidant Assays

#### 2.4.1. 1,1-Diphenyl-2-picrylhydrazyl (DPPH) Radical Scavenging Activity

**Figure 4:**
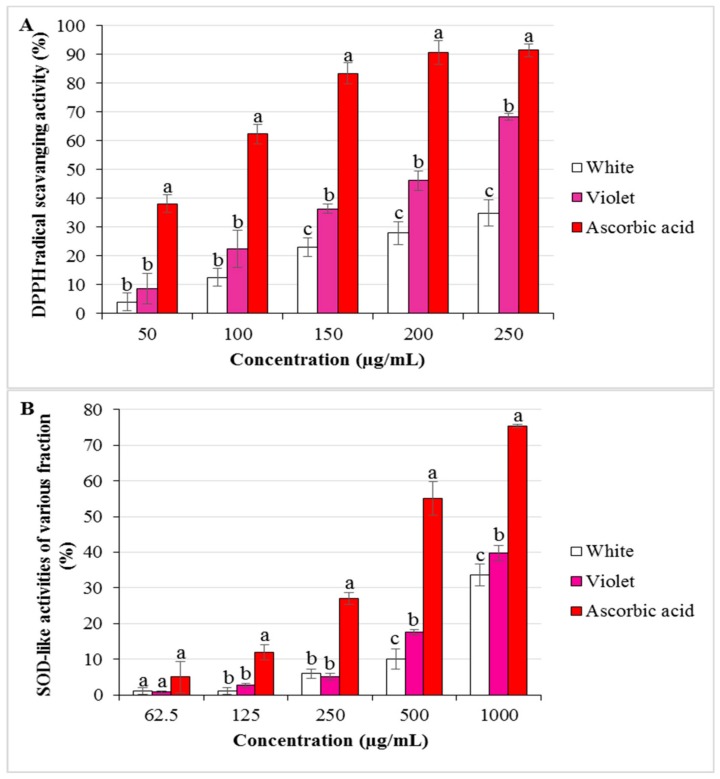
(**A**) 1,1-Diphenyl-2-picrylhydrazyl (DPPH) radical scavenging activity of ethanol extracts from white and violet flowers. (**B**) Superoxide radical scavenging activity in ethanol extracts from white and violet flowers, respectively. Shown are the mean values (in per cent) of triplicated experiments ± standard deviation (SD). Different letters (a, b, c, respectively) indicate a significant difference at *p* < 0.05, applying a Duncan’s multiple range test.

DPPH radical scavenging activity was measured using ethanol extracts from two different flowers at different concentrations (50 to 250 μg/mL; Figure 4A). DPPH scavenging activity depended on the extract concentrations, in both flowers. In particular, the highest DPPH radical scavenging activity was observed in violet flowers (68.45%) and an extract concentration of 250 μg/mL, followed by white flowers (34.91%) at the same concentration. Moreover, violet flowers also showed higher radical scavenging activities than white flowers at lower concentrations (100 to 200 μg/mL). However, at concentrations below 100 μg/mL, the enzymatic activities did not differ between flowers.

#### 2.4.2. Dismutase-Like Activity

Superoxide dismutase (SOD)-like activity was determined using extracts from two different flowers (at concentrations from 62.5 to 1000 μg/mL; Figure 4B). Superoxide radical scavenging activity was higher in violet flowers (39.81%) than in white flowers (33.59%), at 1000 μg/mL, each. Furthermore, at a lower concentration of 500 µg/L, violet flowers showed higher enzymatic activity (17.57%) than white flowers (11.51%). In contrast, at concentrations below 500 μg/mL, SOD-like activities did not differ between white and violet flowers.

### 2.5. 3-(4,5-Dimethylthiazol-2-yl)-2,5-diphenyltetrazolium Bromide (MTT) Assay for Cell Viability

Cytotoxic effects of ethanol extracts of white and violet flowers, were determined using an MTT assay. Extracts of white and violet flowers at different concentrations (1.953125 to 1000 μg/mL) were added to RAW 264.7 cells stimulated by a lipopolysaccharide (LPS) treatment. After an incubation period of 24 h, cells treated with violet flowers at a concentration above 3.90625 μg/mL showed a decrease in viability (Figure S5). In contrast, the white flower treatment only reduced cell viability at extract concentrations above 31.25 μg/mL. Therefore, a nitric oxide (NO) assay was performed using extracts of white and violet flowers at concentrations above 3.90625 μg/mL.

### 2.6. Nitric Oxide (NO) Assay

**Figure 5:**
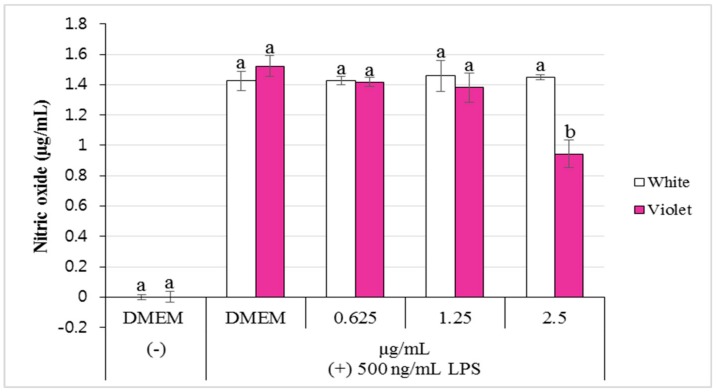
Effect of ethanol extracts from white and violet flowers, respectively, on NO production in LPS-activated RAW264.7 cells. Cells were treated extracts of various concentrations (0.625 to 2.5 µg/mL). The amount of NO in the culture medium was measured using a NO assay. Shown are the mean values (in per cent) of triplicated experiments ± SD. Different letters (a, b) indicate a significant difference at *p* < 0.05, applying a Duncan’s Multiple Range Test (DMRT).

We tested the inhibition of NO accumulation in LPS-activated RAW264.7 cells following treatment with ethanol extracts of white and violet flowers. Cells were treated simultaneously with LPS (500 ng/mL) and ethanol extracts of each flower, at different concentrations (0.625 to 2.5 μg/mL). The production of NO production was measured as nitrite quantity in the culture medium. In a control experiment, a Dulbecco’s Modified Eagle’s medium (DMEM) treatment without LPS activation did not lead to NO production, however, NO was produced when cells were simultaneously treated with LPS (500 ng/mL; 1.52 ± 0.07; 1.43 ± 0.06, respectively). The violet flower extract (at 2.5 µl/mL) significantly inhibited the nitrite production in LPS-activated-RAW 264.7 cells, compared to the control (*p* < 0.05). White flower extract at the same concentration, however, did not inhibit nitrite production (Figure 5). 

In the present study, we found three flavonoids (rutin, quercetin, and kaempferol), and four phenolic acids (4-hydroxybenzoic acid, chlorogenic acid, caffeic acid, *p*-coumaric acid) in white and violet flowers, using LC-MS and HPLC. Previous studies found rutin in flower extracts of *Magnolia denudata* [32], chlorogenic acid, and coumaric acid in the foliage of *Magnolia sieboldii* K. Koch [33], and quercetin and rutin in *Magnolia obovata* leaves [34]. Furthermore, Porter et al. (2015) reported the presence of kaempferol in Magnolia tepals [35]. Among the identified compounds, rutin, which is the most abundant compound, has only recently been introduced in medicinal products and nutritional supplements due to its diverse biological activities, including anti-diabetic, anti-oxidant, anti-inflammatory, and anti-asthmatic properties [36,37,38,39,40]. Similarly, chlorogenic acid, the second most abundant compound, has been widely used in the cosmetic and food processing industries because of physiological effects, e.g. anti-inflammatory, anti-oxidant, and anti-carcinogenic functions [41]. Furthermore, the consumption of quercetin and kaempferol in tea has been shown to enhance antioxidant capacity and to inhibit carcinogen-induced tumors in mice and rats [42]. Therefore, white flowers of *M. denudata* and violet flowers of *M. liliiflora* may be potentially useful raw materials for the production of herbal remedies, food, and cosmetics.

Possible variations between the results reported by the present study and those reported by previous studies are not surprising. This might because ecological factors, including altitude, latitude, longitude, longitude, annual sunshine duration, and annual average temperature, can affect the quality and quantity of phytochemicals [43]. Differences in the extraction methods, solvents, and conditions have led to variations in phytochemical composition [44]. Additionally, environmental factors can affect chemical composition [45]. 

Our results confirm that internal pools of primary metabolites may be correlated with the amounts of secondary metabolites, which is in agreement with the results of a previous study [46]. Carbohydrates play an important role in plant secondary metabolism as metabolic precursors and energy sources. Consistent with the results of the present study, previous studies have shown that the composition and relative concentration of carbon sources affected flavonoid production in callus culture of *Hydrocotyle bonariensis* [47]. The total concentrations of carbohydrates, including glycerol, fructose, galactose, glucose, mannose, mannitol, sucrose, maltose, trehalose, raffinose, and xylose were higher in violet flowers, than that in white flowers, indicating a higher demand for carbon and energy for the production of phenolic compounds. Furthermore, Pearson’s correlation analysis and a HCA showed a significant positive correlation between the concentrations of carbohydrates and phenolic compounds (Figure S2 and Figure 2). Particularly, the higher concentration of sucrose might be required for the higher production of phenolic compounds in violet flowers. Similarly, Zulak et al. (2008) reported that in poppy (*Papaver somniferum*) cell cultures, supplementation with a fungal elicitor promoted the production of alkaloids and thereby rapidly depleted internal sugar reservoirs [48].

Previously, Payyavula et al. (2013) reported that potatoes with red or purple flesh contained higher content of phenolics and anthocyanins, as well as higher sucrose and glucose content than potatoes with white or yellow flesh. Furthermore, sucrose treatment can significantly increase the expression of the phenylpropanoid-related genes *phenylalanine ammonia lase* (*PAL*), *chalcone synthase* (*CHS*), *flavanone 3-hydroxylase* (*F3H*), *dihydroflavonol 4-reductase* (*DFR*), *UDPG flavonoid O-glucosyltransferase* (*UFGT*), *anthocyanin methyltransferase* (*AOMT3*), transcription factors anthocyanin1 (*AN1*), *basic*-*Helix*-*Loop*-*Helix1* (*bHLH1*), WD40, and the expression of the sugar metabolism-related genes *sucrose synthase1* (*SUSY1*), *sucrose synthase4* (*SUSY4*), and *invertase2* (*INV2*), and increase the amounts of phenolic compounds in potato plantlets [49]. Similarly, an increase in sucrose concentration enhances cell growth and phenylpropanoid production in grapevine cell cultures, with the phenylpropanoid production partly correlated with the expression of the phenylpropanoid-related genes *PAL*, *CHS*, and *chalcone*-*flavanone isomerase* (*CHI*) [50]. Park et al. (2016) reported that the exogenous sucrose supply to hairy root cultures of *Scutellaria baicalensis* enhanced the production of flavonoids, such as wogonin, baicalin, and baicalein [51]. 

In our study, we identified bioactive compounds, such as rutin, quercetin, kaempferol, 4-hydroxybenzoic acid, chlorogenic acid, caffeic acid, *p*-coumaric acid, sinapic acid, quinic acid, and vanillic acid by HPLC and GC-TOFMS. Phenolics, including phenolic acids and flavonoids, are considered natural antioxidants due to their ability to donate electrons to oxidative molecules [52,53]. Among the identified flavonoids, rutin, quercetin, and kaempferol have been shown to be potential antioxidants [54]. Comparably, hydrocinnamic acids (chlorogenic acid, caffeic acid, *p*-coumaric acid, sinapic acid) and hydroxybenzoic acids (4-hydroxybenzoic acid, vanillic acid) are known for their antioxidative potential as well [55,56]. Anthocyanins, which belong to the class of flavonoids, are strong natural antioxidants [57]. The present study demonstrates that higher scavenging activities and anti-inflammatory activity can be observed in violet flowers compared to white flowers, using DPPH, SOD-like, and NO activity assays. These antioxidative properties may be due to the higher concentrations of phenolic compounds (which are also natural antioxidants) in violet flowers. The findings are consistent with a previous study reporting that in radish (*Raphanus sativus*), the red variety showed higher antioxidant activity than white radish [58]. Moreover, in lettuce (*Lactuca sativa*), red varieties also contain higher amounts of total phenolics, flavonoids, anthocyanins, and carotenoids, and thus also showed higher antioxidant activities than green lettuce [59].

According to the World Health Organization (WHO), approximately 80% of the global population depends on medicinal plants for their primary healthcare needs [60,61]. Thus, the present study provides evidence for the potential of various applications of white flowers of *M. denudata* and violet flowers of *M. liliiflora* in herbal medicine. Furthermore, further studies should investigate identification of the unknown compound.

## 3. Materials and Methods 

### 3.1. Plant Materials

Ten grams of white flowers of *M. denudata* and violet flowers of *M. liliiflora* were harvested in triplicate from the experimental plantation of Chungnam National University in March 2018, and were immediately submerged in liquid nitrogen (−196 °C). Subsequently, flower samples were freeze-dried at −80°C for three days, and then ground by hand into a fine powder, using mortar and pestle.

### 3.2. LC-MS Analysis for the Quantification of Phenylpropanoid Contents

For the LC-MS of white flowers of *M. denudata*, an Agilent 1200 series HPLC device (Agilent Technologies, Palo Alto, CA, USA) coupled to a model G1315D diode array detector (DAD), and a 4000 Qtrap LC/MS/MS system (Applied Biosystems Instrument, Foster City, CA, USA) using the negative ion mode ([M − H]^−^). The LC-MS conditions were set as follows: scan time of 4.80 s; scan range from 100 to 1300 *m*/*z*; curtain gas (N_2_) at 20.00 psi; nebulizer gas at 50 psi; drying gas at 50 psi; drying gas temperature of 550 °C; entrance potential of 10 V; declustering potential of 100 V; and ionization voltage of 5000 V.

### 3.3. Phenylpropanoid HPLC Analysis

Phenylpropanoid extraction and HPLC analysis were performed for both flower types, according to the method described by Park et al. (2017) [62]. A total of 0.1 g of the sample material was aliquoted into a 15 mL tube, and 2 mL of methanol (80% *v*/*v*) was added. Subsequently, the tube was sonicated for 1 h at 35 °C, and then centrifuged at 4000 rpm for 10 min. The supernatant was collected in a new tube. This procedure was also applied to the remaining sample material. The collected products were dried under nitrogen gas, and subsequently resuspended in 2 mL of methanol. The HPLC analysis system, condition, and gradient program were performed as detailed in a previous study [62]. The separting solvent consisted of a mixture of solvent A (acetic acid/water (0.2:99.8, *v*/*v*)), and solvent B (methanol). Samples were eluted with the following gradient condtions: 0 min, 95% A; 4 min, 95%–85% A; 9 min, 85% A; 14 min, 85%–80% A; 24 min, 80% A; 54 min, 80%–70% A; 55 min, 70%–55% A; 65 min, 55% A; 75 min, 55%–44% A; 77.0 min, 44%–40% A; 79 min, 40% A; 80 min, 40%–20% A; 90 min, 20% A; 91.0 min, 20%–95% A; and 98.0 min, 95% A. The detection wavelength and oven temperature set at 280 nm and 40 °C. The flow rate and injection volume were 1.0 mL/min and 20 μL, respectively. Comparison of retention times and spiking tests were used to identify each peak. Quantitation was performed using the respective calibration curves. The HPLC data was analyzed using SAS software (version 9.4, 2013; SAS Institute, Inc., Cary, NC, USA), applying an Analysis of Variance (ANOVA) evaluation and a Duncan’s Multiple Range Test (DMRT). The level of statistical significance was set at *p* < 0.05.

### 3.4. Total Phenolics

Powdered sample material (200 mg) of white and violet flowers, respectively, was extracted with 2 mL of ethanol and sonicated for 2 h. After an incubation period of 24 h, the extracts were centrifuged at 10,000 rpm for 15 min, and subsequently filtered through a 0.45 µm polyterafluoroethylene (PTFE) hydrophilic syringe filter into a vial. The extracts were then prepared for measuring total phenolic and anthocyanin content. The Folin-Ciocalteu method was used for the quantification of total phenolic content [58]. A total of 100 μL of extract was added to a 15-mL tube containing 3.4 mL of deionized water (DW) and 0.5 mL of 2 N Folin & Ciocalteu’s phenol reagent (Sigma-Aldrich, Yongin, Korea). After incubation for 3 min at 28 °C, 2 mL of sodium carbonate (20%, *w*/*v*) was added to the mixture, which was then incubated for 1 h in the dark. The absorbance of each sample was measured at 760 nm in a standard spectrophotometer (UV). A calibration curve equivalent (standard curve equation: y = 0.001981712x + 0.032496678, R² = 0.997201606) was created using different concentrations (10, 60, 100, 200, 400, 600, 800, and 1000 μg/mL) of a gallic acid standard. The final results are presented as milligrams of gallic acid equivalent per gram of dry weight (mg GAE/g dry weight). 

### 3.5. Total Anthocyanin Content 

The pH differential method, consisting of KCl buffer (0.025 M, pH 1.0) and CH_3_COONa buffer (0.4 M, pH 4.5), was used to measure the total anthocyanin content in the prepared crude extract of each sample [59]. Aliquots of 1 mL of the extract were mixed with 4 mL of each of the buffers, and incubated at 28 °C for 15 min for equilibration. The absorbance reading was performed at 510 and 700 nm against DW as a blank control. The absorbance (A) of each sample was calculated with the following equation: A = [(A510 − A700) pH 1.0 − (A510 − A700) pH 4.5]. The total anthocyanin concentration (%, *w*/*w*) was then determined using the following formula: monomeric anthocyanin pigment (mg/L) = (A × MW × DF × 1000)/(ε × l); where the molecular weight (MW) = 449.2 g.mol^−1^, and the extinction coefficient ε = 26,900 M in L mol^−1^cm^−1^ of cyanidin-3-glucoside; the factor 1000 converts g to mg; DF indicates the dilution factor; l indicates the cuvette pathlength. The final results were converted to milligrams of cyanidin-3-glucoside equivalents per gram dry weight (mg CGE/g dry weight).

### 3.6. GC-TOFMS Analysis

The extraction method described by Li et al. (2014) [63] was used to measure hydrophilic metabolite contents in white and violet flowers. To 10 mg powdered samples we added 1 mL of a water‒chloroform‒methanol solution (1:1:2.5 *v*/*v*/*v*), then 60 µL of ribitol (0.2 mg/mL) was added as an internal standard (IS). The mixture was incubated and mixed in a compact thermomixer at 1200 rpm and 37 °C for 30 min, followed by centrifugation at 9000 rpm for 10 min. Subsequently, 0.8 mL of the polar phase was moved into a new 2 mL tube containing 0.4 mL of deionized water. This mixture was again centrifuged at 9000 rpm for 5 min. A CVE-2000 centrifugal concentrator (Eyela, Japan) and a FD8512 freeze-dryer (Ilshin Lab Co., Ltd., Dongducheon, Korea) were used to facilitate evaporation of the methanol‒water phase. The residues, containing hydrophilic metabolites were subjected to a two-stage process, methoxime derivatization and trimethylsilyl etherification. A total of 80 μL of methoxyamine hydrochloride in pyridine (20 mg/mL) was added to each sample, which was then incubated under constant shaking at 30 °C for 90 min. Then, 80 μL of *N*-methyl-*N*-(trimethylsilyl)trifluoroacetamide was added, followed by another incubation period at 37 °C for 30 min. GC-TOFMS analysis was carried out exactly as descried by Li et al., 2014 [63]. The split ratio was set at 1:25. The injector temperature and flow rate of helium through the column were 230 °C and 1.0 mL/min, respectively.The temperature program was established as follows: initial temperature of 80 °C for 2 min, followed by an increase to 320 °C at 15 °C/min, and a 10 min hold at 320 °C. The ion-source temperatures were set at 200 °C, and the transfer line was 250. The detector voltage and scanned mass range were 1700 V. T and 85−600 *m*/*z*, respectively. The quantitaion of each analyte was based on the peak area ratio relative to that of the IS (Figure S6 and Table S1). The GC-TOFMS analysis was performed in three technical replicates. A Statistical Analysis System software (SAS, system 9.4, 2013; SAS Institute, Inc., Cary, NC, USA) and Multi Experiment Viewer (version 4.4.0, Dana-Farber Cancer Institute, Boston, MA, USA, http://www.tm4.org/mev/) were used for the analysis of the GC-TOFMS raw data. A principal component analyses (PCA) was performed on the correlation matrices and represented mean ± standard deviation of the technical replicates. 

### 3.7. Superoxide Dismutase (SOD)-Like Activity

SOD-like activity of extracts of white and violet flowers was evaluated as previously described [64]. A total of 100 µL of the extract solution (62.5–1000 µg/mL) was placed in a test tube. To this, 120 µL of 50 mM Tris-HCl buffer (pH 8.5) and 20 µL of 7.2 mM pyrogallol solution were added, followed by incubation at 25 °C for 10 min. Then, 40 µL of hydroxylamine hydrochloride (1 M) was added to stop the reaction. The absorbance was measured at 420 nm using a standard UV spectrophotometer. A blank control containing distilled water instead of sample extracts was used, and ascorbic acid was used as a standard. 

The following formula was applied: Superoxide dismutase-like activity (%) = [(1 − A1)/A0] × 100, where A0 is the absorbance of the control, and A1 is the absorbance of the sample. All samples were analyzed in three technical replicates. 

### 3.8. DPPH Assay

DPPH radical scavenging activity in extracts of different colored flowers was measured according to the method described in Park et al. (2018) [59]. A total of 100 µL of extract solution (62.5–1000 µg/mL) was placed in a test tube. To this, 100 µL of 1 mM DPPH was added, following incubation at 25 °C for 10 min in the dark. The absorbance was read at 520 nm using a UV spectrophotometer. A blank control containing distilled water instead of sample extracts was used, and ascorbic acid was used as a standard. The following formula was used: DPPH radical scavenging activity (%) = [(1 – A1)/A0] × 100, where A0 is the absorbance of the control and A1 is the absorbance of the sample. All samples were analyzed in three technical replicates.

### 3.9. MTT Assay for Cell Viability

A MTT assay was performed as described previously [65]. RAW 264.7 cells (3 × 10^4^ cells/well) were plated in 96-well plates and cultured for 24 h, followed by supplementation with LPS (50 ng/mL) and ethanol extracts at different concentrations (1.953125–15.625 µg/mL). After the second incubation period of 24 h, 10 µL of MTT were supplemented to the medium, and cells were again and cultured for 4 h. After this, the supernatant was discarded and the dimethyl sulfoxide was used for the dissolution of the emerged formazan crystals. Absorbance was measured at 540 nm. The percentage of dead cells was measured relative to the control sample, which was a treatment with DMEM, instead of LPS or ethanol extracts. Nitrite quantity was determined using a sodium nitrite standard curve.

### 3.10. Nitric Oxide (NO) Assay

An NO assay was performed as described previously [65]. RAW 264.7 cells were cultured in a modification of Eagle’s medium after Dulbecco, and supplemented with 1% antibiotic–antimycotic and 10% fetal bovine serum, under 5% CO_2_ at 37 °C. To investigate the effect of crude extracts of white and violet flowers, on NO production, cells were loaded onto 96-well plates (3 × 10^4^ cells/well) using fresh culture medium, and were then pre-incubated for 2 h. After this, LPS (50 ng/mL) and sample extracts (62.5–1000 µg/mL) were added, followed by another incubation period of 24 h. Subsequently, the nitrite quantity was measured as an indicator of NO production. In brief, 100 μL of Griess reagent (0.1% *N*-(1-naphthyl) ethylenediamine dihydrochloride and 1% sulfanilamide in 2.5% phosphoric acid) was added to 100 μL of cell culture medium. Then, the mixture was incubated for 10 min at room temperature, after which absorbance was measured at 540 nm using a microplate reader. Fresh medium was used as a blank control in every experiment. The amount of nitrite was determined using a sodium nitrite standard curve.

### 3.11. Statistical Analysis

Data from GC-TOFMS and HPLC were analyzed using Statistical Analysis System (SAS, system 9.4, 2013; SAS Institute, Inc., Cary, NC, USA); SIMCA-P (version 12.0, Umetrics, Umeå, Sweden), and Multi Experiment Viewer (version 4.4.0, Dana-Farber Cancer Institute, Boston, MA, USA, http://www.tm4.org/mev/) software. Results are presented as mean ± standard deviation of three technical replicates. Data obtained from GC-TOFMS was scaled to unit variance scaling and then subjected to partial least-squares discriminant analysis (PLS-DA) using SIMCA-P software (version 12.0, Umetrics, Umeå, Sweden) to determine differences in metabolite profiles between the varieties. The PLS-DA output is presented as score plot to demonstrate contrasts. Furthermore, Pearson’s correlation analysis was performed based on the relative concentrations of 42 metabolites applying a standardization procedure in SAS 9.4. The resulting correlation coefficients were visualized by a hierarchical clustering analysis (HCA), and a heat map which was produced from the Multi Experiment Viewer, and Duncan’s multiple range test (DMRT, *p* < 0.05) was performed to depict a metabolic linkage map.

## 4. Conclusions

Here, we report the relationship between primary and secondary metabolites in the different colored Magnolia flowers. Violet flowers contained higher concentrations of phenolic compounds and of carbohydrates, compared to white flowers. This may reflect the higher carbon and energy demand for the synthesis of phenylpropanoids. The higher amounts of phenolic compounds in violet flowers may partially explain higher radical scavenging activity and anti-inflammatory properties. Correspondingly, our results confirm that HPLC- and GC-TOFMS-based metabolomic profiling are suitable methods to determine the interplay between primary and secondary metabolites in *M. denudata* and *M. liliiflora* flowers.

## Figures and Tables

**Table 1:** Phenylpropanoid contents (mg/g dry wt.) of white flowers of *M. denudata* and violet flowers of *M. liliiflora*.

No.	Retention Time	Phenolics	[M − H]^−^ (*m*/*z*)	White	Violet
**1**	20.98	4-hydroxybenzoic acid	137.6	0.27 ± 0.02	1.20 ± 0.46 *
**2**	22.85	Chlorogenic acid	353.8	0.47 ± 0.10	1.69 ± 0.42 *
**3**	27.78	Caffeic acid	179.9	tr ^1^	0.04 ± 0.00 *
**4**	41.71	*p*-coumaric acid	163.4	tr	0.26 ± 0.04 *
**5**	61.99	Rutin	609.0	10.47 ± 0.34	13.62 ± 1.45 *
**6**	73.66	Quercetin	301.1	0.38 ± 0.00	0.37 ± 0.00
**7**	80.36	Kaempferol	285.5	0.12 ± 0.00	0.13 ± 0.02
		Total		11.71 ± 0.46	17.31 ± 2.39 *

^1^ tr: trace. Asterisks indicate significant differences (Student’s *t*-test, * *p* < 0.05).

**Table 2:** Total polyphenolic and anthocyanin contents in ethanol extracts of white flowers of *M. denudata* and violet flowers of *M. liliiflora*.

	Total Phenolics (mg/g)	Total Anthocyanin (mg/g)
Violet Flowers	16.94 ± 0.71 *	0.78 ± 0.16 *
White Flowers	14.07 ± 0.55	0.21 ± 0.08

Asterisks indicate significant differences (Student’s *t*-test, * *p* < 0.05).

## Supplementary Materials

The following are available online. Figure S1: LC-MS spectrum of phenolic compounds identified in white flowers of *Magnolia denudata* Desr. 1,4-hydroxybenzoic acid; 2, Chlorogenic acid; 3, Caffeic acid; 4, *p*-coumaric acid; 5, Rutin; 6, Quercetin; 7, Kaempferol. Figure S2: Scores and loading plots of principal components 1 and 2 of the PCA results obtained from polar metabolite data of white flowers of *M. denudata* and violet flowers of *M. liliiflora*. 1–44, the compounds listed in Table S1. Figure S3: Correlation matrix of 10 phenolics from white flowers of *M. denudata* and violet flowers of *M. liliiflora*. Each square indicates the Pearson’s correlation coefficient of a pair of compounds, with the correlation coefficient indicated by the intensity of the red and blue colors. Figure S4: Metabolite peak height differences of white flowers of *Magnolia denudata* and violet flowers of *Magnolia liliiflora* based on Duncan’s Multiple Range Test (*p* < 0.05) using GC-TOFMS. Figure S5: Effect of ethanol extracts from white and violet flowers, respectively, on the viability of RAW 264.7 cells. Cells were treated with different concentrations (1.953125 to 1000 µg/mL) of extracts of white and violet flowers. Cell viability was measured by an MTT assay. Figure S6: Selected ion chromatograms of hydrophilic metabolites extracted white flowers of *Magnolia denudata* as MO/TMS derivatives separated on a 30 m × 0.25 mm i.d. fused silica capillary column coated with 0.25 μm CP-SIL 8 CB low bleed. The numbers represent the same compounds as for Table 1. Table S1: Metabolites identified in GC-TOFMS chromatograms of white flowers of *Magnolia denudata*.
