# Supplementary material for: Analysis of Metabolites in White Flowers of Magnolia Denudata Desr. and Violet Flowers of Magnolia Liliiflora Desr
Source: Molecules. 2018 Jun 27;23(7):1558. doi: 10.3390/molecules23071558 (PMC6100211; doi:10.3390/molecules23071558)
Supplement: Supplementary file 1 [file molecules-23-01558-s001.pdf]

molecules

Analysis of Metabolites in White Flowers of *Magnolia Denudata* Desr. and Violet Flowers of *Magnolia Liliiflora* Desr.

Chang Ha Park [1], Soo-Yun Park [2], Sook Young Lee [3], Jae Kwang Kim [4,*] and Sang Un Park [1,*]

[1] Department of Crop Science, Chungnam National University, 99 Daehak-Ro, Yuseong-gu, Daejeon 34134, Korea; parkch804@gmail.com
[2] National Institute of Agricultural Sciences, Rural Development Administration, Wanju-gun, Jeonbuk 54875, Korea; psy22@korea.kr
[3] Regional Innovation Center for Dental Science & Engineering, Chosun University, 309 Pilmun-daero, Dong-gu, Gwangju 501-759, Korea; koreanseedbank@gmail.com
[4] Division of Life Sciences and Bio-Resource and Environmental Center, Incheon National University, Incheon 406-772, Korea
* Correspondence: kjkpj@inu.ac.kr (J.K.K.); supark@cnu.ac.kr (S.U.P.); Tel.: +82-32-835-8241 (J.K.K.); +82-42-821-6730 (S.U.P.); Fax: +82-32-835-0763 (J.K.K.); +82-42-822-2631 (S.U.P.)

Received: 27 May 2018; Accepted: 21 June 2018; Published: date

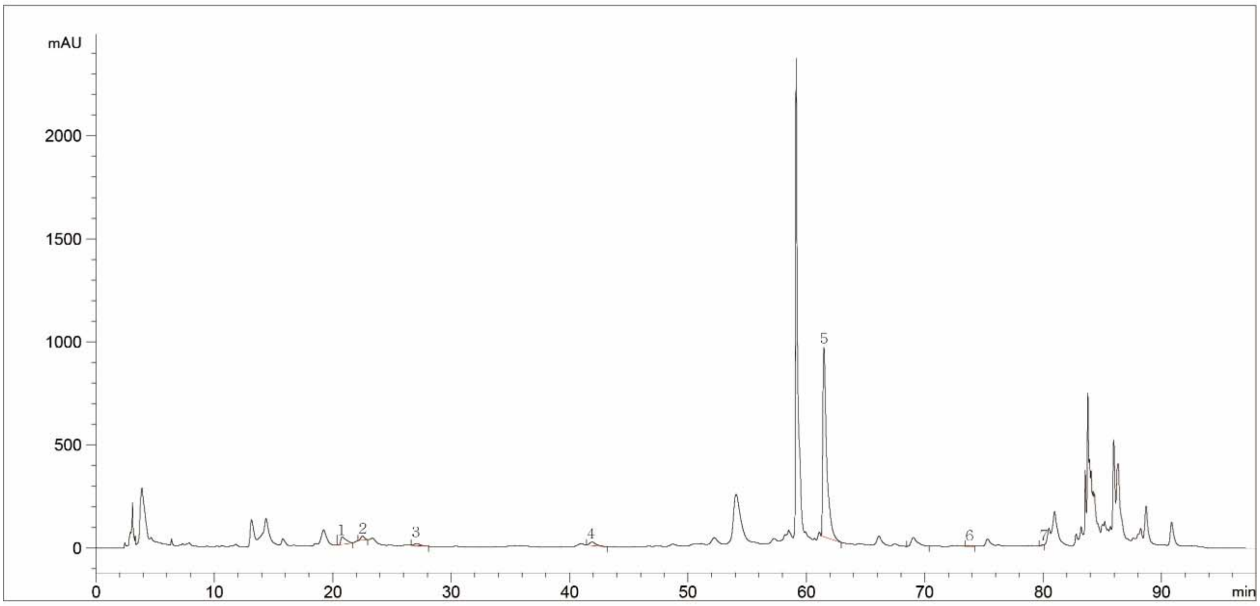

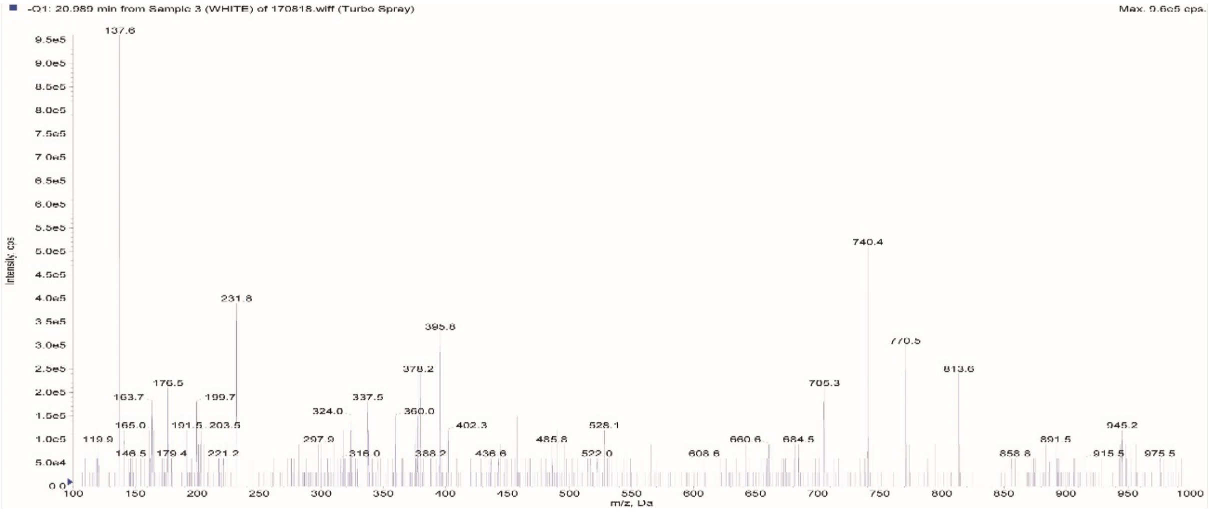
-Q1: 20.989 min from Sample 3 (WHITE) of 170818.wiff (Turbo Spray)
Max. 9.6e5 cps.
Intensity, cps
m/z, Da
137.6
740.4
231.8
395.8
770.5
813.6
378.2
705.3
176.5
163.7
199.7
337.5
324.0
360.0
945.2
402.3
528.1
165.0
191.5
203.5
119.9
297.9
485.8
660.6
684.5
891.5
146.5
179.4
221.2
318.0
388.2
436.6
522.0
608.6
858.8
915.5
975.5

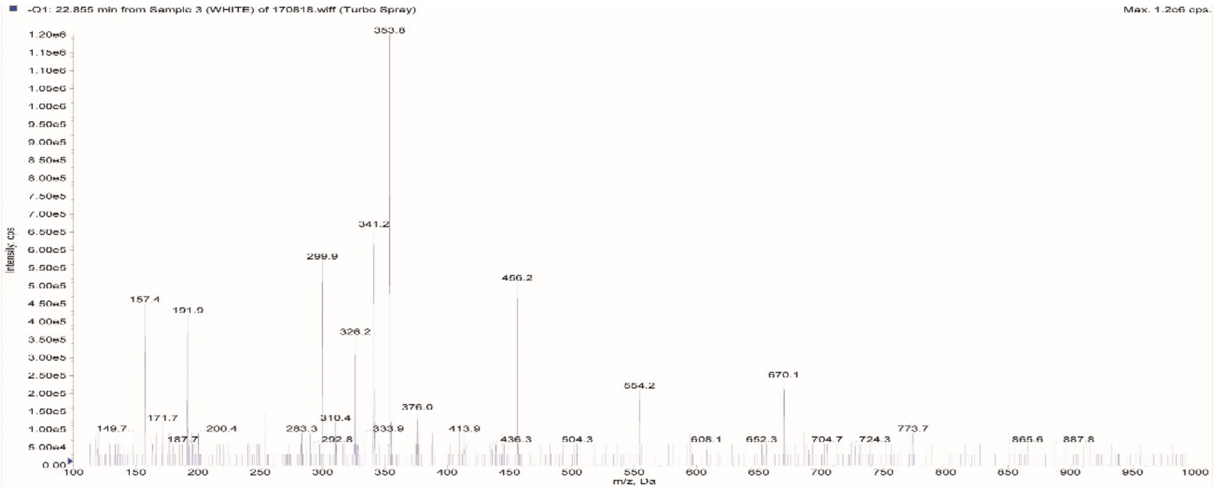
-Q1: 22.855 min from Sample 3 (WHITE) of 170818.wiff (Turbo Spray)
Max. 1.2e6 cps.
Intensity, cps
m/z, Da
353.8
341.2
299.9
456.2
157.4
191.9
326.2
670.1
554.2
376.0
171.7
310.4
773.7
149.7
200.4
283.3
333.9
413.9
187.7
292.8
436.3
504.3
608.1
652.3
704.7
724.3
865.6
887.8

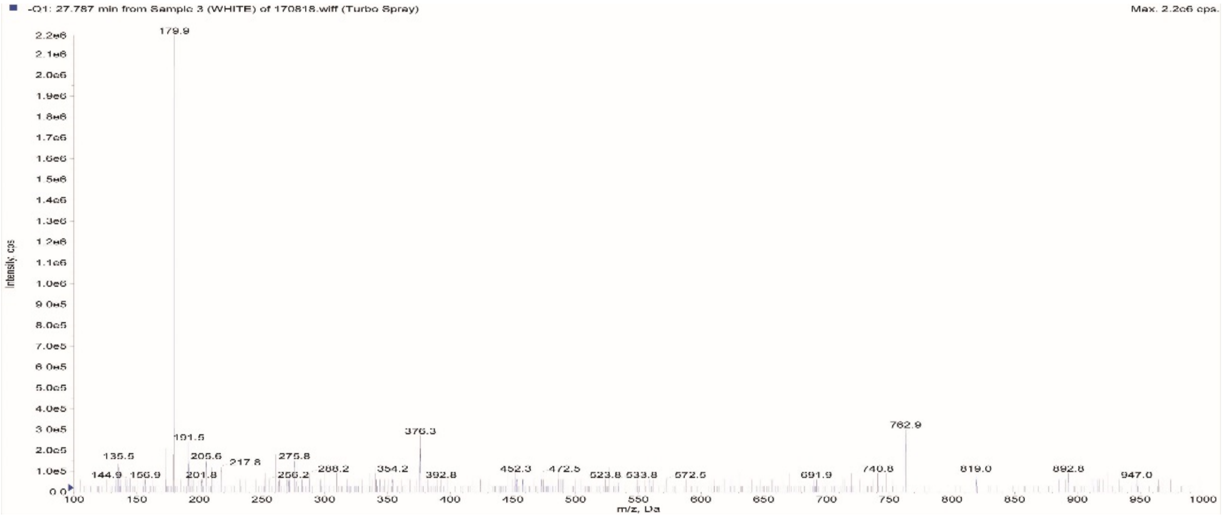
-Q1: 27.787 min from Sample 3 (WHITE) of 170818.wiff (Turbo Spray)
Max. 2.2e6 cps.
Intensity, cps
m/z, Da
179.9
762.9
376.3
191.5
135.5
205.6
275.8
217.8
288.2
354.2
452.3
472.5
740.8
819.0
892.8
144.9
156.9
201.8
256.2
392.8
523.8
533.8
572.5
691.9
947.0

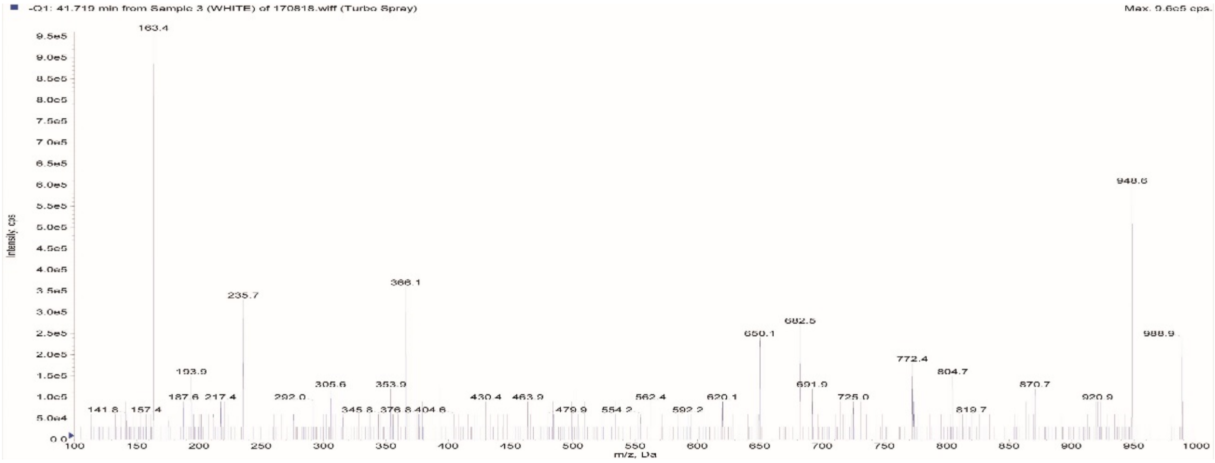
-Q1: 41.719 min from Sample 3 (WHITE) of 170818.wiff (Turbo Spray)
Max. 9.6e5 cps.
Intensity, cps
m/z, Da
163.4
948.6
366.1
235.7
682.5
650.1
988.9
772.4
193.9
804.7
305.6
353.9
691.9
870.7
187.6
217.4
292.0
430.4
463.9
562.4
620.1
725.0
920.9
141.8
157.4
345.8
376.8
404.6
479.9
554.2
592.2
819.7

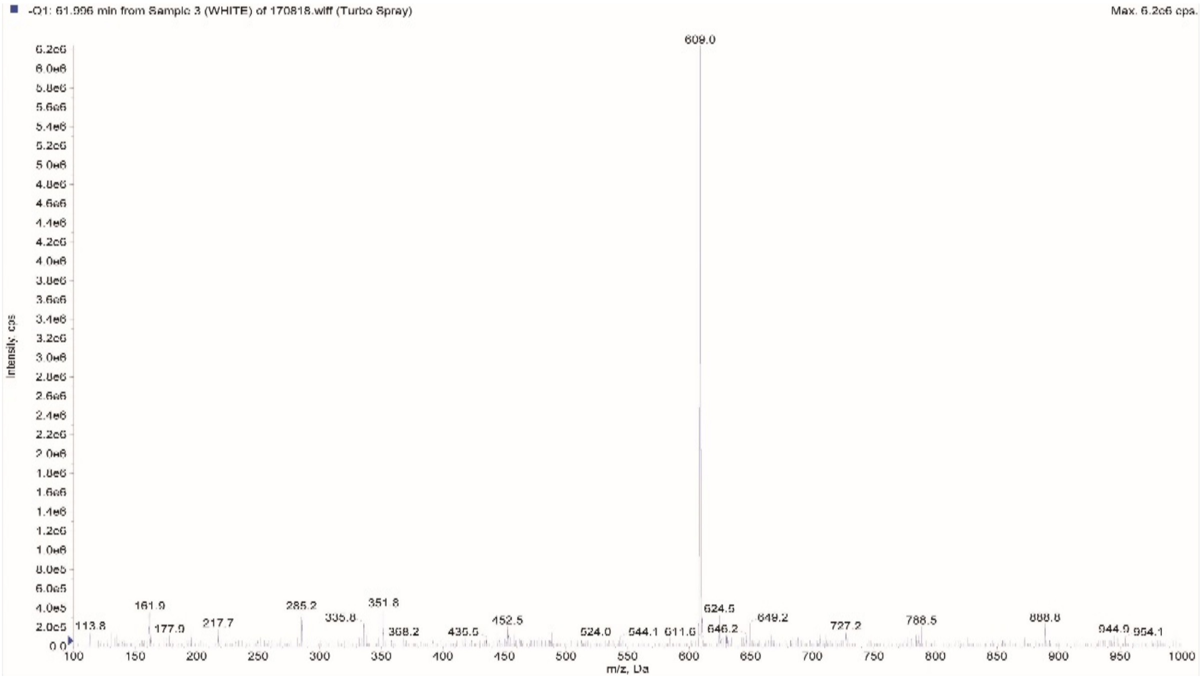
-Q1: 61.996 min from Sample 3 (WHITE) of 170818.wiff (Turbo Spray)
Max. 6.2e6 cps.
Intensity, cps
m/z, Da
609.0
161.9
285.2
351.8
624.5
113.8
177.9
217.7
335.8
368.2
435.5
452.5
524.0
544.1
611.6
646.2
649.2
727.2
788.5
888.8
944.9
954.1

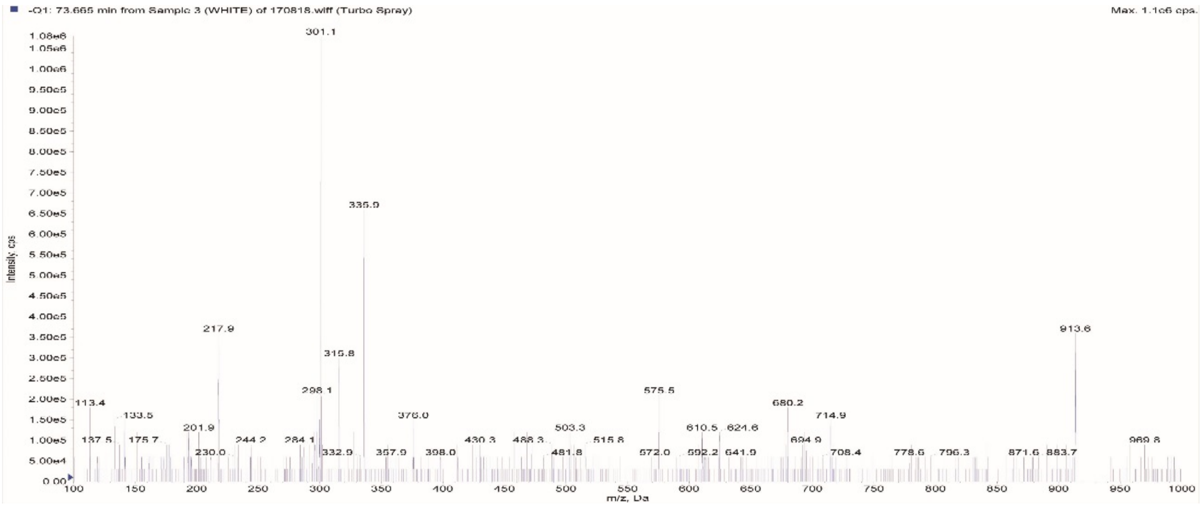
-Q1: 73.665 min from Sample 3 (WHITE) of 170818.wiff (Turbo Spray)
Max. 1.1e6 cps.
Intensity, cps
m/z, Da
301.1
335.9
217.9
913.6
315.8
298.1
575.5
113.4
680.2
133.5
376.0
714.9
201.9
503.3
610.5
624.6
137.5
175.7
230.0
244.2
284.1
332.9
357.9
398.0
430.3
488.3
481.8
515.8
572.0
592.2
641.9
694.9
708.4
778.6
796.3
871.6
883.7
969.8

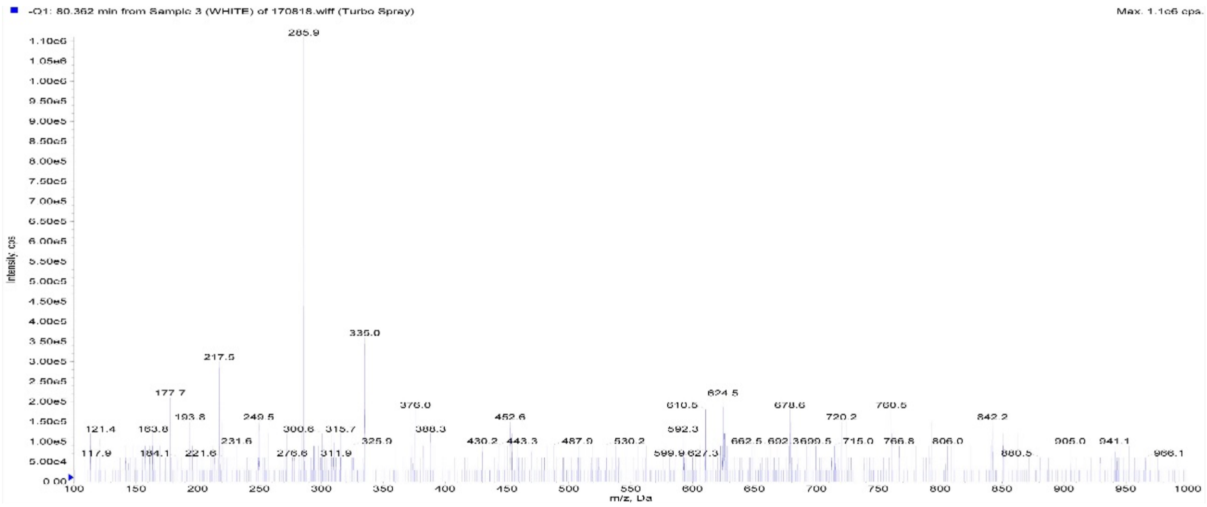

Figure S1. LC-MS spectrum of phenolic compounds identified in white flowers of *Magnolia denudate* Desr. 1, 4-hydroxybenzoic acid; 2, Chlorogenic acid; 3, Caffeic acid; 4, *p*-coumaric acid; 5, Rutin; 6, Quercetin; 7, Kaempferol.

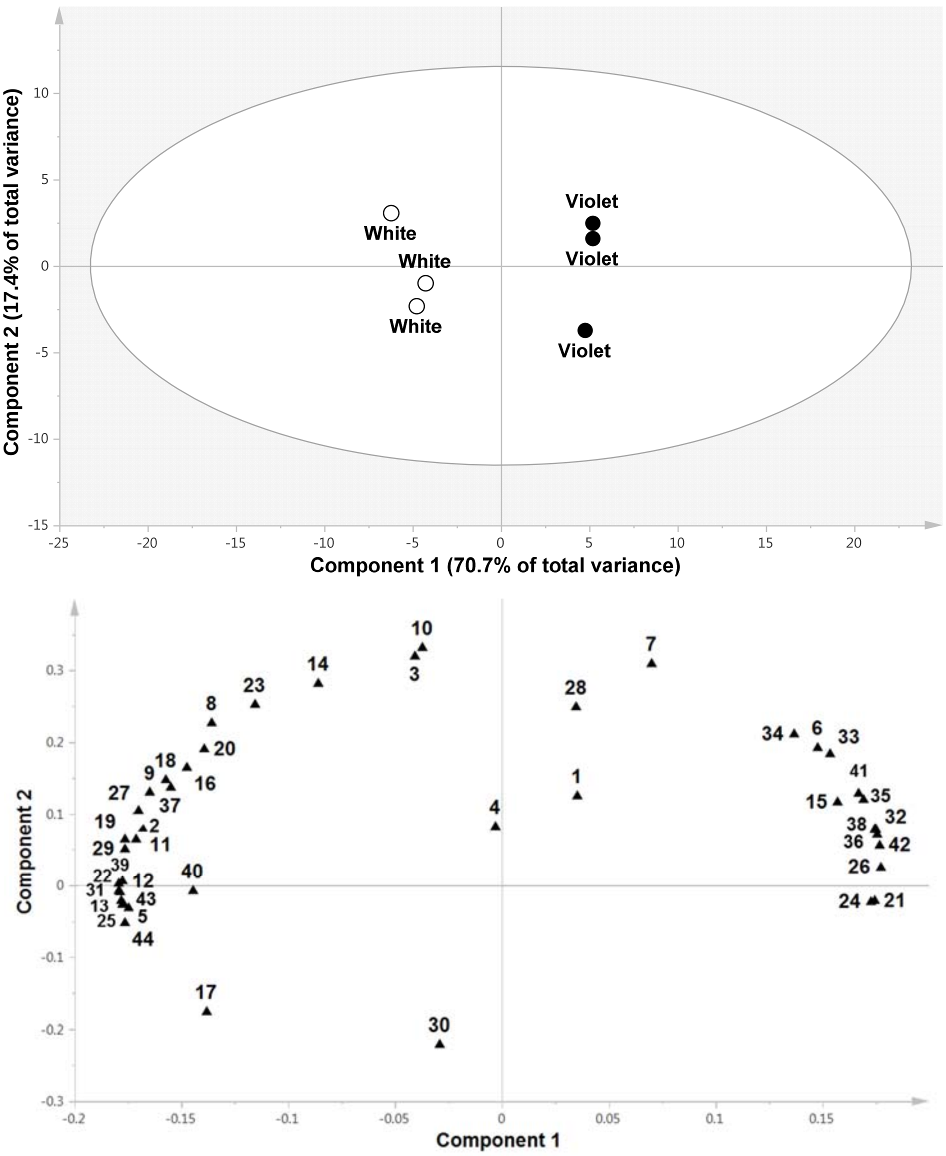

Figure S2. Scores and loading plots of principal components 1 and 2 of the PCA results obtained from polar metabolite data of white flowers of *M. denudate* and violet flowers of *M. liliiflora*. 1–44, the compounds listed in Table S1.

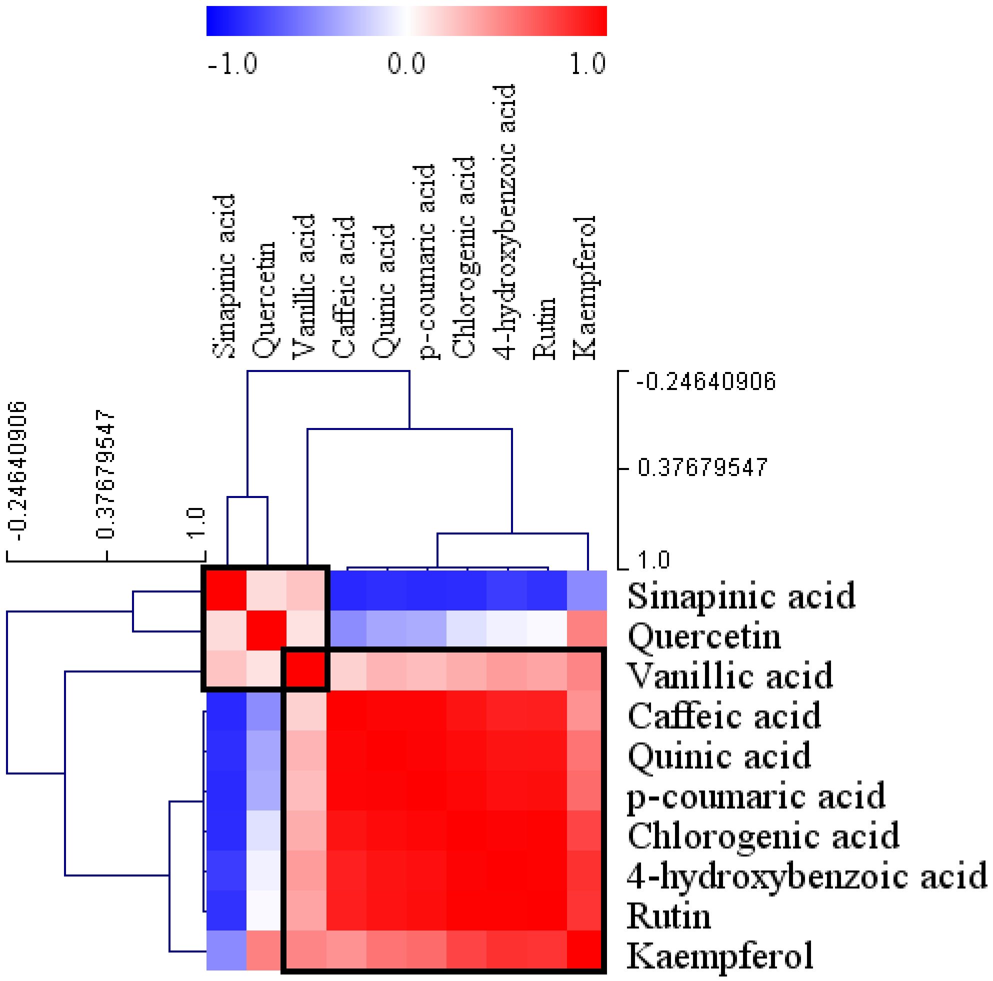

Figure S3. Correlation matrix of 10 phenolics from white flowers of *M. denudata* and violet flowers of *M. liliiflora*. Each square indicates the Pearson's correlation coefficient of a pair of compounds, with the correlation coefficient indicated by the intensity of the red and blue colors.

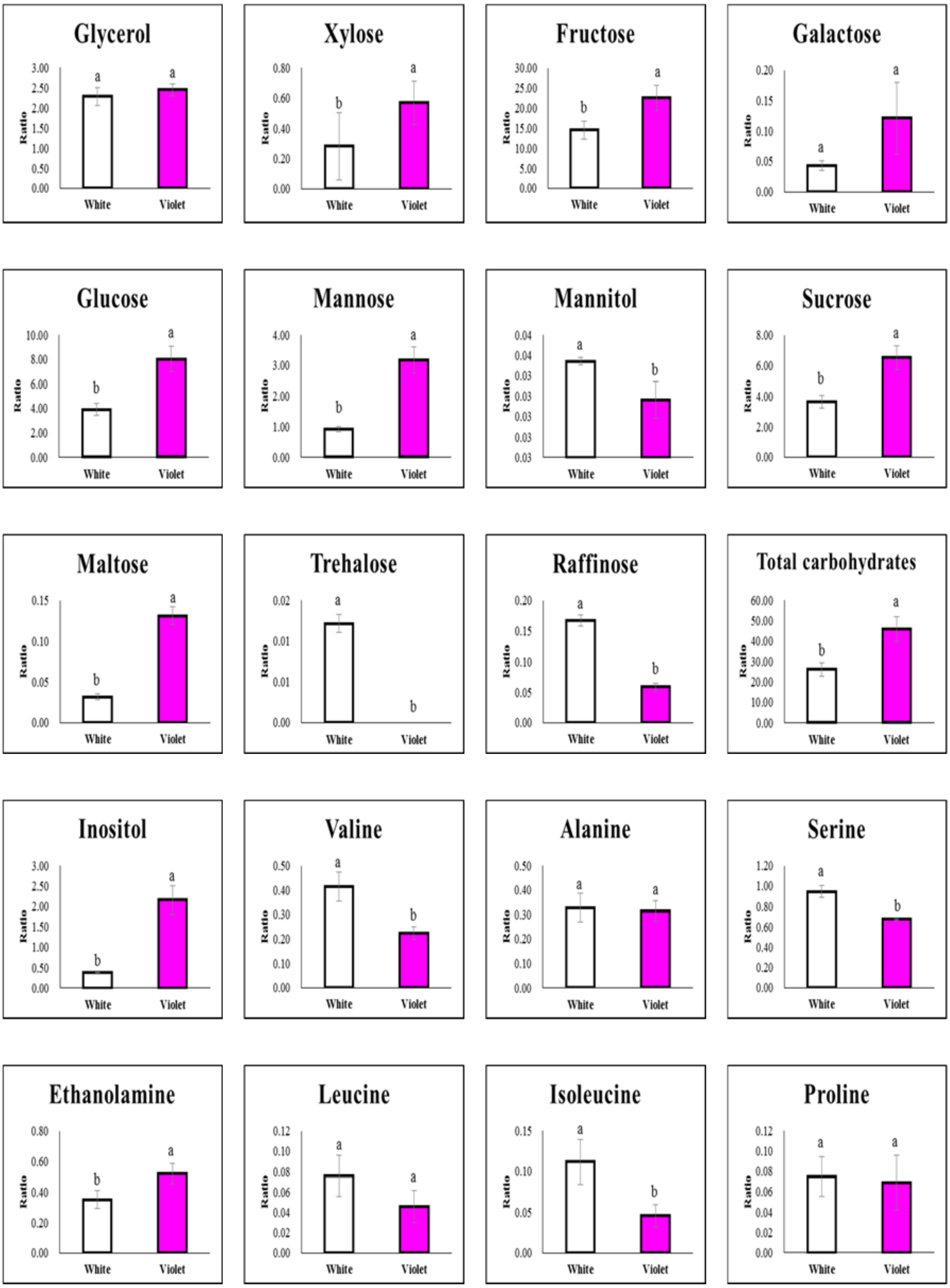
Glycerol
3.00
2.50
2.00
1.50
1.00
0.50
0.00
Ratio
a
a
White
Violet
Xylose
0.80
0.60
0.40
0.20
0.00
Ratio
b
a
White
Violet
Fructose
30.00
25.00
20.00
15.00
10.00
5.00
0.00
Ratio
b
a
White
Violet
Galactose
0.20
0.15
0.10
0.05
0.00
Ratio
a
a
White
Violet
Glucose
10.00
8.00
6.00
4.00
2.00
0.00
Ratio
b
a
White
Violet
Mannose
4.00
3.00
2.00
1.00
0.00
Ratio
b
a
White
Violet
Mannitol
0.04
0.04
0.03
0.03
0.03
0.03
0.03
Ratio
a
b
White
Violet
Sucrose
8.00
6.00
4.00
2.00
0.00
Ratio
b
a
White
Violet
Maltose
0.15
0.10
0.05
0.00
Ratio
b
a
White
Violet
Trehalose
0.02
0.01
0.01
0.00
Ratio
a
b
White
Violet
Raffinose
0.20
0.15
0.10
0.05
0.00
Ratio
a
b
White
Violet
Total carbohydrates
60.00
50.00
40.00
30.00
20.00
10.00
0.00
Ratio
b
a
White
Violet
Inositol
3.00
2.50
2.00
1.50
1.00
0.50
0.00
Ratio
b
a
White
Violet
Valine
0.50
0.40
0.30
0.20
0.10
0.00
Ratio
a
b
White
Violet
Alanine
0.50
0.40
0.30
0.20
0.10
0.00
Ratio
a
a
White
Violet
Serine
1.20
1.00
0.80
0.60
0.40
0.20
0.00
Ratio
a
b
White
Violet
Ethanolamine
0.80
0.60
0.40
0.20
0.00
Ratio
b
a
White
Violet
Leucine
0.12
0.10
0.08
0.06
0.04
0.02
0.00
Ratio
a
a
White
Violet
Isoleucine
0.15
0.10
0.05
0.00
Ratio
a
b
White
Violet
Proline
0.12
0.10
0.08
0.06
0.04
0.02
0.00
Ratio
a
a
White
Violet

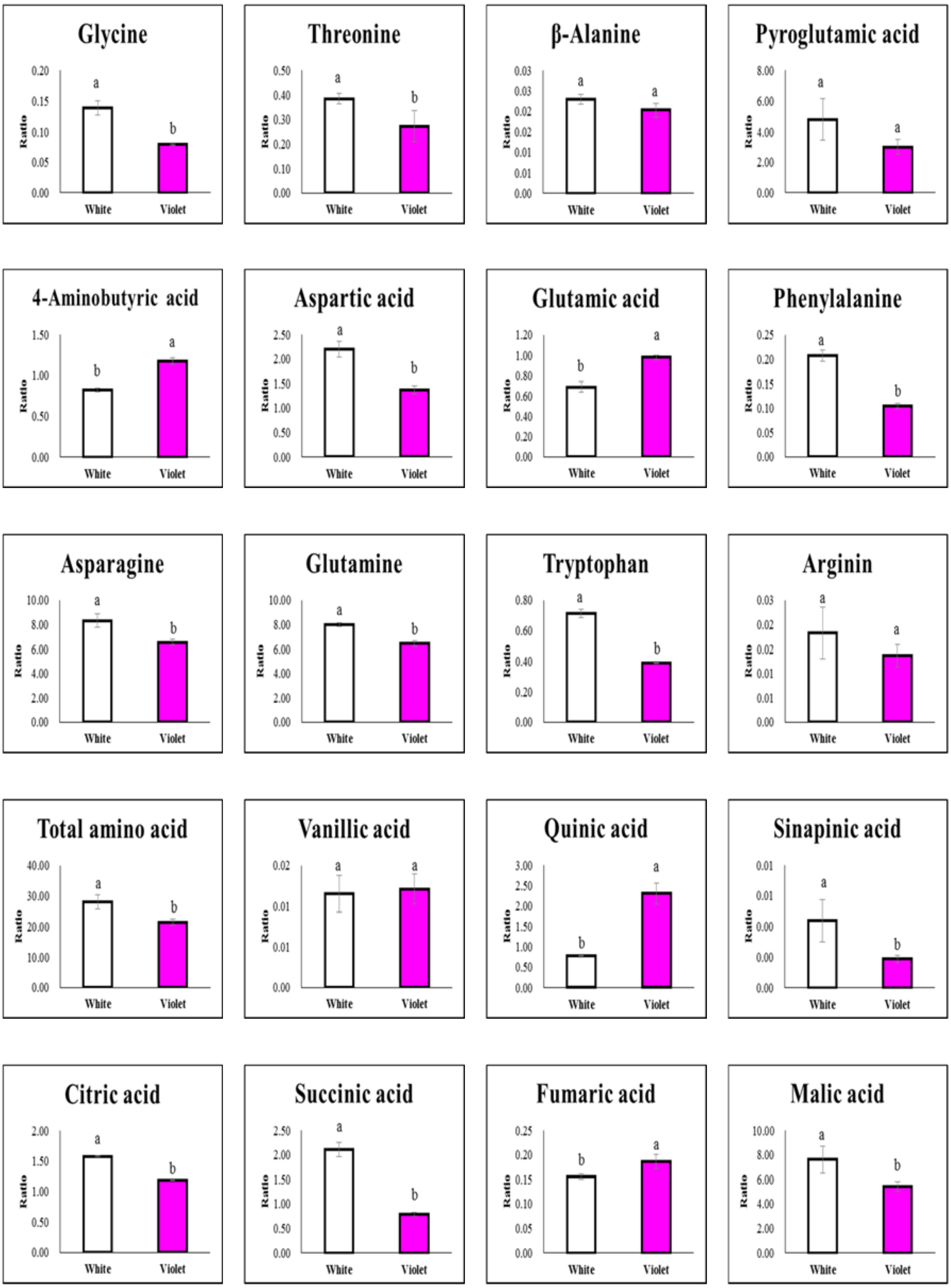
Glycine
0.20 0.15 0.10 0.05 0.00
Ratio
a b
White Violet
Threonine
0.50 0.40 0.30 0.20 0.10 0.00
Ratio
a b
White Violet
β-Alanine
0.03 0.03 0.02 0.02 0.01 0.01 0.00
Ratio
a a
White Violet
Pyroglutamic acid
8.00 6.00 4.00 2.00 0.00
Ratio
a a
White Violet
4-Aminobutyric acid
1.50 1.00 0.50 0.00
Ratio
b a
White Violet
Aspartic acid
2.50 2.00 1.50 1.00 0.50 0.00
Ratio
a b
White Violet
Glutamic acid
1.20 1.00 0.80 0.60 0.40 0.20 0.00
Ratio
b a
White Violet
Phenylalanine
0.25 0.20 0.15 0.10 0.05 0.00
Ratio
a b
White Violet
Asparagine
10.00 8.00 6.00 4.00 2.00 0.00
Ratio
a b
White Violet
Glutamine
10.00 8.00 6.00 4.00 2.00 0.00
Ratio
a b
White Violet
Tryptophan
0.80 0.60 0.40 0.20 0.00
Ratio
a b
White Violet
Arginin
0.03 0.02 0.02 0.01 0.01 0.00
Ratio
a a
White Violet
Total amino acid
40.00 30.00 20.00 10.00 0.00
Ratio
a b
White Violet
Vanillic acid
0.02 0.01 0.01 0.00
Ratio
a a
White Violet
Quinic acid
3.00 2.50 2.00 1.50 1.00 0.50 0.00
Ratio
b a
White Violet
Sinapinic acid
0.01 0.01 0.00 0.00 0.00
Ratio
a b
White Violet
Citric acid
2.00 1.50 1.00 0.50 0.00
Ratio
a b
White Violet
Succinic acid
2.50 2.00 1.50 1.00 0.50 0.00
Ratio
a b
White Violet
Fumaric acid
0.25 0.20 0.15 0.10 0.05 0.00
Ratio
b a
White Violet
Malic acid
10.00 8.00 6.00 4.00 2.00 0.00
Ratio
a b
White Violet

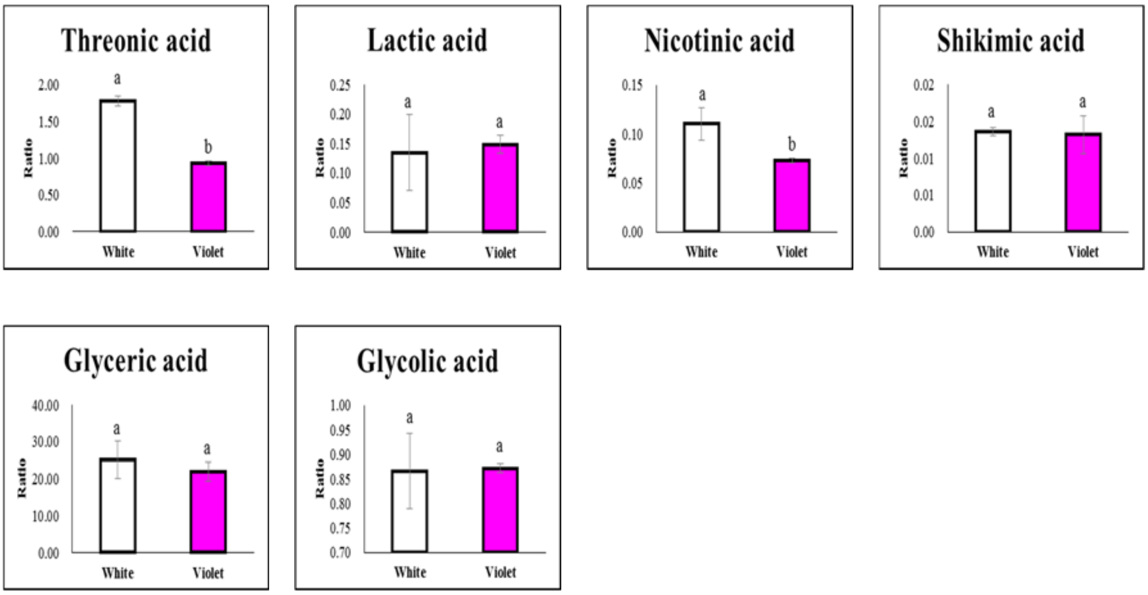

Figure S4. Metabolite peak height differences of white flowers of *Magnolia denudate* and violet flowers of *Magnolia liliiflora* based on Duncan's Multiple Range Test ($p < 0.05$) using GC–TOFMS.

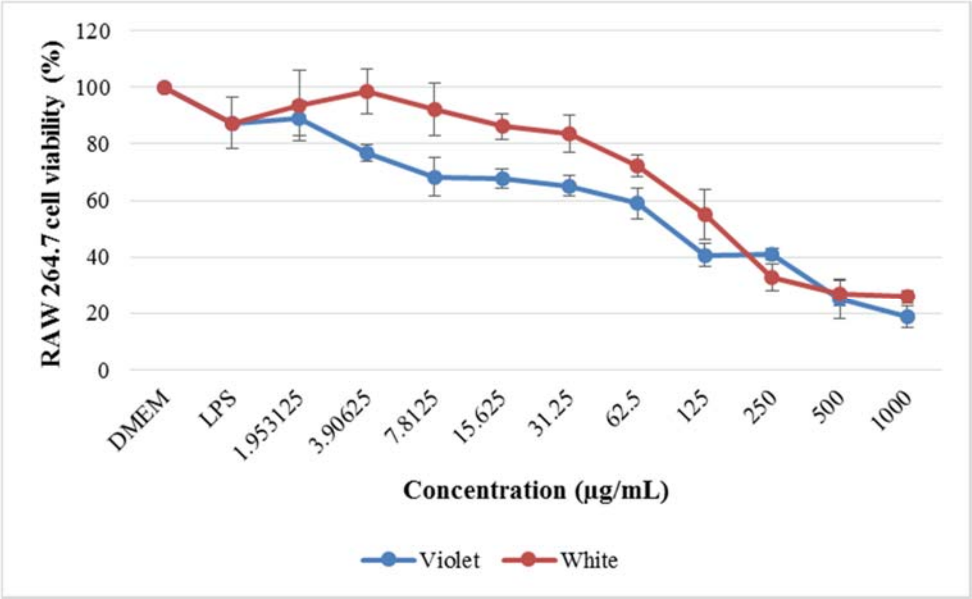

Figure S5. Effect of ethanol extracts from white and violet flowers, respectively, on the viability of RAW 264.7 cells. Cells were treated with different concentrations (1.953125 to 1,000 μg/mL) of extracts of white and violet flowers. Cell viability was measured by a 3-(4,5-dimethylthiazol-2-yl)-2,5-diphenyltetrazolium bromide (MTT) assay.

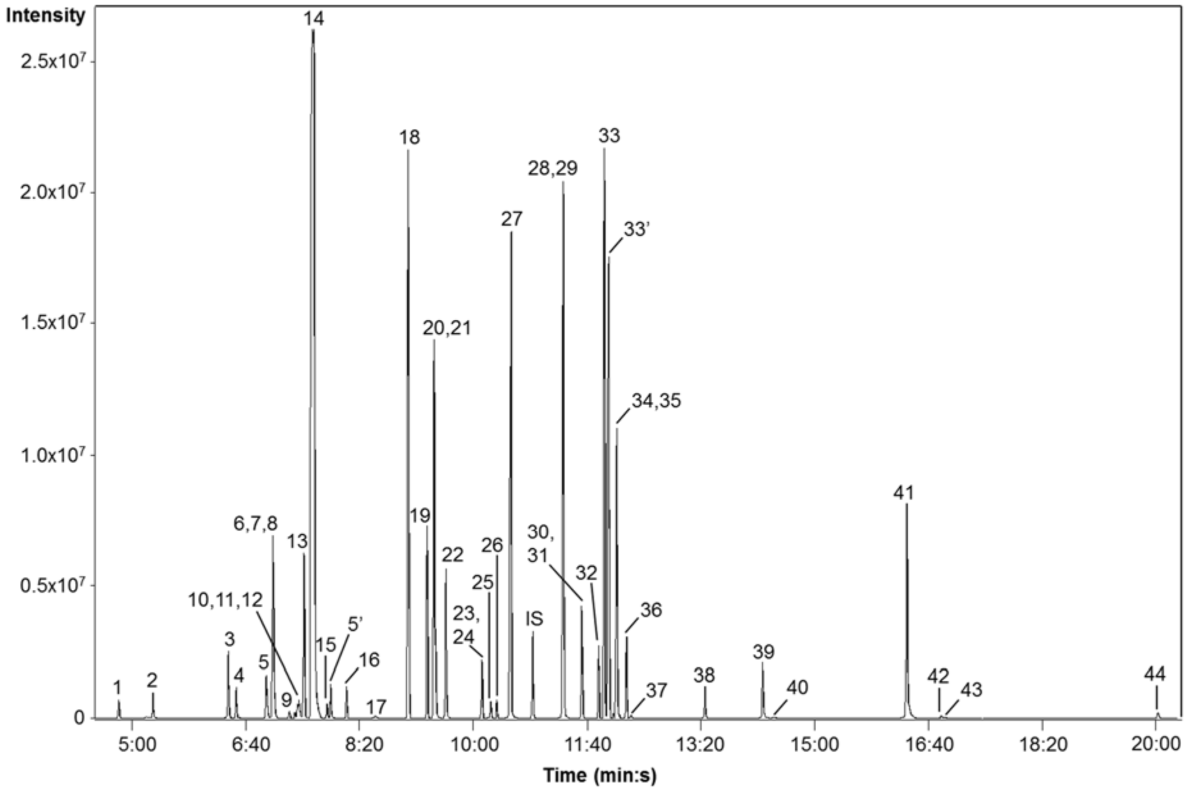

Figure S6. Selected ion chromatograms of hydrophilic metabolites extracted white flowers of *Magnolia denudate* as MO/TMS derivatives separated on a 30 m × 0.25 mm i.d. fused silica capillary column coated with 0.25 µm CP-SIL 8 CB low bleed. The numbers represent the same compounds as for Table 1.

Table S1. Metabolites identified in GC-TOFMS chromatograms of white flowers of *Magnolia denudate*.

No.[1)]	Compound	RT[2)]	RRT[3)]	Selected ion for quantification[4)]
1	Lactic acid	4.810	0.443	147
2	Alanine	5.310	0.489	146
3	Glycolic acid	6.413	0.590	147
4	Valine	6.530	0.601	146
5	Serine	6.973	0.641	116
6	Ethanolamine	7.053	0.649	174
7	Glycerol	7.072	0.651	147
8	Leucine	7.095	0.653	158
9	Isoleucine	7.308	0.672	158
10	Proline	7.392	0.680	142
11	Nicotinic acid	7.427	0.683	180
12	Glycine	7.452	0.686	174
13	Succinic acid	7.525	0.692	147
14	Glyceric acid	7.667	0.705	147
15	Fumaric acid	7.865	0.724	245
16	Threonine	8.147	0.749	219
17	*β*-Alanine	8.570	0.788	174
18	Malic acid	9.050	0.833	147
19	Aspartic acid	9.327	0.858	100
20	Pyroglutamic acid	9.423	0.867	156
21	4-Aminobutyric acid	9.450	0.869	174
22	Threonic acid	9.598	0.883	147
23	Arginine	10.097	0.929	142
24	Glutamic acid	10.132	0.932	246
25	Phenylalanine	10.262	0.944	218
26	Xylose	10.347	0.952	103
27	Asparagine	10.555	0.971	116
28	Ribitol (Internal Standard)	10.870	1.000	217
IS	Vanillic acid	11.290	1.039	297
29	Glutamine	11.322	1.042	156
30	Shikimic acid	11.455	1.054	227
31	Citric acid	11.592	1.066	273
32	Quinic acid	11.842	1.089	345
33	Fructose	11.920	1.097	103
34	Galactose	12.067	1.110	147
35	Glucose	12.102	1.113	147
36	Mannose	12.245	1.126	147
37	Mannitol	12.315	1.133	319
38	Inositol	13.393	1.232	305
39	Tryptophan	14.242	1.310	202
40	Sinapic acid	14.397	1.324	338
41	Sucrose	16.352	1.504	217
42	Maltose	16.860	1.551	147
43	Trehalose	16.913	1.556	191
44	Raffinose	20.032	1.843	217

[1)]Numbers represent the compound index for the chromatogram peaks shown in Figure S5. [2)]Retention time (min). [3)]Relative retention time (retention time of the analyte/retention time of the IS). [4)]Specific mass ion used for quantification.
